# Dietary Sources of Anthocyanins and Their Association with Metabolome Biomarkers and Cardiometabolic Risk Factors in an Observational Study

**DOI:** 10.3390/nu15051208

**Published:** 2023-02-28

**Authors:** Hamza Mostafa, Tomás Meroño, Antonio Miñarro, Alex Sánchez-Pla, Fabián Lanuza, Raul Zamora-Ros, Agnetha Linn Rostgaard-Hansen, Núria Estanyol-Torres, Marta Cubedo-Culleré, Anne Tjønneland, Rikard Landberg, Jytte Halkjær, Cristina Andres-Lacueva

**Affiliations:** 1 Biomarkers and Nutrimetabolomics Laboratory, Department of Nutrition, Food Sciences and Gastronomy, Xarxa d’Innovació Alimentària (XIA), Nutrition and Food Safety Research Institute (INSA), Facultat de Farmàcia i Ciències de l’Alimentació, Universitat de Barcelona (UB), 08028 Barcelona, Spain; 2 Centro de Investigación Biomédica en Red de Fragilidad y Envejecimiento Saludable (CIBERFES), Instituto de Salud Carlos III, 28029 Madrid, Spain; 3 Departament de Genètica, Microbiologia i Estadística, Facultat de Biologia, Universitat de Barcelona (UB), 08028 Barcelona, Spain; 4 Unit of Nutrition and Cancer, Cancer Epidemiology Research Program, Catalan Institute of Oncology (ICO), Bellvitge Biomedical Research Institute (IDIBELL), 08908 L’Hospitalet de Llobregat, Spain; 5 Danish Cancer Society Research Center, Strandboulevarden 49, DK 2100 Copenhagen, Denmark; 6 Department of Biology and Biological Engineering, Division of Food and Nutrition Science, Chalmers University of Technology, 412 96 Gothenburg, Sweden

**Keywords:** metabolomics, anthocyanins, food matrix, diet, gut microbiota, berries, cardiometabolic health

## Abstract

Anthocyanins (ACNs) are (poly)phenols associated with reduced cardiometabolic risk. Associations between dietary intake, microbial metabolism, and cardiometabolic health benefits of ACNs have not been fully characterized. Our aims were to study the association between ACN intake, considering its dietary sources, and plasma metabolites, and to relate them with cardiometabolic risk factors in an observational study. A total of 1351 samples from 624 participants (55% female, mean age: 45 ± 12 years old) enrolled in the DCH-NG MAX study were studied using a targeted metabolomic analysis. Twenty-four-hour dietary recalls were used to collect dietary data at baseline, six, and twelve months. ACN content of foods was calculated using Phenol Explorer and foods were categorized into food groups. The median intake of total ACNs was 1.6mg/day. Using mixed graphical models, ACNs from different foods showed specific associations with plasma metabolome biomarkers. Combining these results with censored regression analysis, metabolites associated with ACNs intake were: salsolinol sulfate, 4-methylcatechol sulfate, linoleoyl carnitine, 3,4-dihydroxyphenylacetic acid, and one valerolactone. Salsolinol sulfate and 4-methylcatechol sulfate, both related to the intake of ACNs mainly from berries, were inversely associated with visceral adipose tissue. In conclusion, plasma metabolome biomarkers of dietary ACNs depended on the dietary source and some of them, such as salsolinol sulfate and 4-methylcatechol sulfate may link berry intake with cardiometabolic health benefits.

## 1. Introduction

Anthocyanins (ACNs) are phytochemical compounds of the subclass of flavonoids in the broader (poly)phenol class, highly present in plant foods, such as berries, grapes, eggplants, and many other colored fruits and vegetables [1,2]. Most of the dietary ACNs reach the large intestine unaffected where they may affect both gut microbial composition and microbial metabolism of ACNs [2,3]. Dietary ACNs and their microbial metabolites are suggested to play roles in the prevention and treatment of cardiometabolic diseases [4,5]. Microbial metabolites of ACNs have been shown to reach higher concentrations in the systemic circulation and may be more bioactive than the consumed ACNs per se [6]. Nonetheless, studies evaluating the association between ACN dietary intake and plasma concentrations of ACN-derived microbial metabolites in observational studies are lacking.

Sources of variability in ACN metabolism by the host and gut microbiota could be related to differences in consumed types and quantity of ACNs, as well as to a food matrix effect. Ultimately, these differences might be translated into different health effects of ACNs in response to their intake. Up to the moment, besides parent ACNs, such as cyanidin, delphinidin, malvidin, and petunidin, various metabolites derived from microbial and host metabolism (i.e., protocatechuic acid, syringic acid, and 4-hydroxybenzoic acid) have been associated with the consumption of berries in dietary intervention studies [7,8,9]. However, to the best of our knowledge, associations between the intake of ACNs, annotated according to their dietary sources, microbial metabolites, and cardiometabolic risk factors have not been studied in an observational study. Our hypothesis is that specific sets of microbial metabolites would be associated with dietary ACNs from different food sources and that these will display differential associations with cardiometabolic risk factors. The aims of this study were to evaluate the association between the intake of ACNs, considering their different dietary sources, and plasma metabolites. The aim was further to explore the associations between ACN-related metabolites and cardiometabolic risk factors in a subsample of the Danish Diet, Cancer, and Health-Next Generations (DCH-NG) MAX study [10].

## 2. Materials and Methods

### 2.1. Study Design and Subjects

We studied a validation subsample within the Diet, Cancer, and Health-Next Generations (DCH-NG) cohort: the DCH-NG MAX study. The DCH-NG was an extension of the previous cohort the Diet, Cancer, and Health (DCH) [10]. A sample of 39,554 participants was included in the DCH-NG involving biological children, their spouses, and grandchildren of the DCH cohort [11]. The DCH-NG MAX study recruited 720 volunteers with residency in Copenhagen, aged 18 years old or more, between August 2017 and January 2019. The major aims of the MAX study were to validate a semi-quantitative food frequency questionnaire against the twenty-four-hour dietary recalls (24-HDR) and to examine the plasma and urine metabolome reproducibility as well as gut microbial stability on a long-term scale. Biological samples, health examinations such as anthropometric and blood pressure measurements, and two questionnaires about lifestyle and dietary habits were collected at baseline, 6, and 12 months.

The DCH-NG cohort study was approved by the Danish Data Protection Agency (journal number 2013-41-2043/2014-231-0094) and by the Committee on Health Research Ethics for the Capital Region of Denmark (journal number H-15001257). The volunteers provided their written informed consent to participate in the study. All the details about clinical measurements, dietary and metabolomics data were previously detailed [11].

### 2.2. Anthropometric Measurements

Participants were asked to wear underwear and be barefoot for measuring height and weight using a wireless stadiometer and a body composition analyzer, respectively (SECA mBCA515, Hamburg, Germany). Height and weight were measured to the nearest 0.1 cm and 0.01 kg, respectively, and body mass index (BMI) was calculated. The waist circumference was measured twice at the midpoint between the lower rib margin and the iliac crest. A third measurement for the waist circumference was measured if the difference between the first two was more than 1 cm. Blood pressure and pulse rate were measured 3 times using the left arm, considering the measurement with the lower systolic blood pressure and its corresponding diastolic blood pressure value as valid. DEXA-validated bioimpedance instrument (SECA mBCA515, Germany) was used to estimate visceral adipose tissue volume.

### 2.3. Dietary Data

The 24-h dietary recalls (24-HDR) were recorded at baseline, 6 and 12 months using a Danish version of the web-based tool myfood24 (www.myfood24.org/) (7 February 2023) from Leeds University [12], containing almost 1600 Danish food items. All foods consumed the day before the examinations were reported by the participants in either grams or in standard portion size. The percentage of calories using the energy equivalents for carbohydrates, proteins, and fat was used to indicate the intake of macronutrients. Complex food products were appointed as recipes or dishes. The McCance and Widdowson’s Food Composition Table [13], or recipes from the food frequency questionnaires in the DCH were used to have the standardized recipes [14].

#### Dietary Intake of Anthocyanins

Estimation of the intake of polyphenols from 24-HDRs was completed by a protocol using “in-house” software developed by the University of Barcelona, the Bellvitge Biomedical Research Institute (IDIBELL), and the Centro de Investigación Biomédica en Red (CIBER) ©. A link between all 24-HDR food items or ingredients and the foods from the Phenol-Explorer database was created [15]. The intake of individual (poly)phenols in mg/day was obtained and ACN consumption from separate foods were estimated as the sum of 71 individual ACNs included in Phenol-Explorer database. The estimated intake of dietary (poly)phenols of the DCH-NG MAX study has previously been described [16]. A total of 147 food items that contain ACNs were used to estimate the total dietary ACN intake as shown in the Supplementary Table S1. Intake of berries was estimated as the sum of foods with at least 50% of its composition or recipe made by berries. These include raw and frozen berries, berries marmalades or jams, and stewed berries. Dietary ACN intake related with the other foods were classified and added up according to the following food groups: dairy products with berries (including ice cream and yogurt), other fruits (i.e., plums, cherries, apples, etc.), non-alcoholic drinks (including fruit smoothies and juices), wines, vegetables, mixed dishes (meat or fish dishes with vegetables containing ACNs), and bakery (including pastry, biscuits, desserts, and waffles with berries or other ACN-containing preparations). (Supplementary Table S2). Intakes of foods not containing ACN were disregarded.

### 2.4. Blood Sampling, Analysis of Cardiometabolic Risk Factors and Metabolomics

Participants were instructed to maintain a fasting time of 1–9 h (mean fasting time: 5 h) during all the examination days. Blood samples were taken into Vacutainer tubes containing lithium heparin at baseline time 0 (*n* = 624), 6 months (*n* = 380), and 12 months (*n* = 349). Within 2 h of blood draw, plasma was obtained by centrifugation, and samples were stored at −80 °C. After that, plasma samples were delivered to the Danish National Biobank (DNB), where plasma was divided into aliquots and sent to University of Barcelona and kept at −80 °C until metabolomic analysis. Other blood measurements such as hemoglobin, A1c (HbA1c), serum lipids, and high sensitivity C reactive protein (hsCRP) were measured as described before [17].

#### 2.4.1. Metabolomics Analysis of Plasma Samples

Repeated measures of the plasma metabolome at all three time points were used for metabolomics analysis. All the samples were prepared and analyzed using the targeted UPLC-MS/MS method described previously, with slight modifications [18,19]. Briefly, 100 µL of plasma was added into protein precipitation plates together with 500 µL cold acetonitrile containing 1.5 M formic acid and 10 mM of ammonium formate and were kept at −20 °C for 10 min to enhance protein precipitation. Then, positive pressure was applied to recover the extracts, which were taken to dryness and reconstituted with 100 µL of an 80:20 *v/v* water:aceto nitrile solution containing 0.1% *v/v* formic acid and 100 ppb of a mixture of 13 internal standards. Samples were then transferred to 96-well plates and analyzed by a targeted metabolomic analysis using an Agilent 1290 Infinity UPLC system coupled to a Sciex QTRAP 6500 mass spectrometer, using the operating conditions described elsewhere [18]. The Sciex OS 2.1.6 software (Sciex, Framingham, MA, USA) was used for data processing.

#### 2.4.2. Metabolomics Data Pre-Processing

The POMA R/Bioconductor package (https://github.com/nutrimetabolomics/POMA) (7 February 2023) was used for the pre-processing of metabolomics data [20]. Metabolites with more than 40% missing values, and those with a coefficient of variation (CV) > 30% in internal quality control were removed. K-nearest neighbor (KNN) algorithm and correction of batch effects using the ComBat function (“sva” R package) were used to impute the remaining missing values [21], while auto-scaling and Euclidean distances (±1.5× Interquartile range) were used to normalize the data and remove the outliers, respectively. The final metabolomics dataset included the concentration of 408 plasma metabolites.

### 2.5. Statistical Analyses

For descriptive statistics, intake of total ACNs (irrespective of their dietary source) was categorized by tertiles using 0.3–8.9 mg as thresholds. Continuous variables following a normal distribution are shown as mean ± SD, and those following a skewed distribution are shown as median (p25–p75). Sociodemographic and clinical characteristics were compared across tertiles of ACNs intake using linear mixed models in a random intercepts model adjusted for age and sex. Associations between intake of ACN dietary sources and their association with cardiometabolic risk factors were tested using linear mixed models in random intercepts models adjusted for age, sex, and BMI.

First, associations between intake of total ACNs and metabolome biomarkers were analyzed using a censored regression for panel data with “censReg” and “plm” R-packages [22]. Censored regression models were applied due to the right-skewed distribution of total ACN intake and the considerable proportion of zero values (24% of non-consumers of ACNs). Covariates included in the models were age, sex, and BMI. *p*-values were adjusted for multiple comparisons using the Benjamini–Hochberg method, and adjusted *p*-values <0.05 were considered statistically significant. Second, associations between ACNs from different dietary sources and metabolites were assessed using Mixed Graphical Models (MGM) with the “mgm” R-package [23]. MGMs are undirected probabilistic graphical models able to represent associations between nodes adjusted for all the other variables in the model. MGM specifications were set to allow the maximum number of interactions in the network. Variables in the model were dietary ACN intakes by food categories (8 food groups), and the whole metabolomics set of variables. The agreement between repeated measurements for total dietary intake of ACNs and for ACN intake from different dietary sources was poor across the study evaluations (intra-class correlation coefficient < 0.15). Therefore, all observations of the study were considered independent and were included in MGM analysis (k = 1351). For visual clarity, only the first-order neighborhood of ACNs food sources was plotted.

To evaluate the associations between metabolites and cardiometabolic risk factors linear mixed models were used in random intercepts models adjusted for age, sex, and BMI. Metabolites were selected based on the combination of both analyses, censored regression, and MGM. Standardized coefficients were plotted in a heatmap built using the “pheatmap” R-package (Kolde R (2019). pheatmap: Pretty Heatmaps).

All statistical analyses were performed using R, version 4.1.3. (R foundation, Austria).

## 3. Results

### 3.1. Sociodemographic, Clinical, and Dietary Characteristics

At baseline, out of the 720 volunteers who agreed to participate in the study, 624 had completed clinical, dietary, and plasma metabolomics data. Of the 624 participants included, 55% were female, aged (mean ± SD) 45 ± 12 years old, and had a BMI of 25 ± 4 kg/m^2^. At 6 months, 380 participants had completed clinical, dietary, and metabolomics data and at 12 months completed data were available for 349 participants. Only, 287 participants had completed clinical dietary and plasma metabolomics data available at all three time points.

The distribution of total ACN intake was right-skewed with a median value of 1.6 (p25–p75: 0.0–26.9) mg/day and a mean value of 26.4 (SD: 60.4) mg/day. Berries were the highest contributors to total ACN intake with a mean contribution of 34%, followed by wines with 33%, and non-alcoholic drinks (which included fruit smoothies and juices) with 20% of the total reported intake. Other fruits (i.e., cherries, apples, and plums) and vegetables were minor contributors with 4% and 2%, respectively. Bakery (pastry, biscuits, and desserts), dairy products (yogurts and strawberry ice creams or ice creams with berries), and other mixed dishes (dishes including vegetables with ACNs) contributed within a similar range between 2 and 3% (Supplementary Table S1).

Participants were divided into tertiles based on the consumed reported intakes of total ACNs as shown in Table 1. There were no significant differences in clinical characteristics across tertiles of ACN intake. Consistently, there were no statistically significant associations between total ACN intake and cardiometabolic risk factors (data not shown). Dietary characteristics are illustrated in Supplementary Table S2. Participants in the highest compared to the ones at the lowest tertile of ACN intake showed statistically significant higher consumption of total protein, saturated fatty acids (SFA), monounsaturated fatty acids (MUFA), polyunsaturated fatty acids (PUFA), alcohol, fruits, and berries.

### 3.2. Association between Intake of ACN Dietary Sources and Cardiometabolic Risk Factors

**Figure 1:**
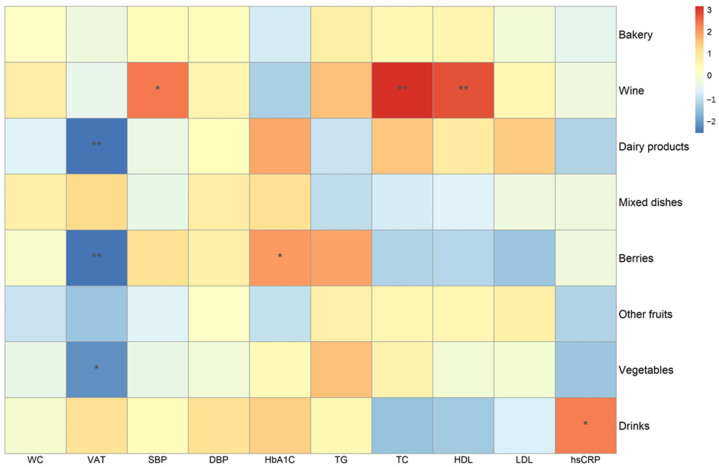
Association between different self-reported food groups and cardiometabolic risk factors in the DCH-NG MAX study (*n* = 624, k = 1351). Standardized coefficients according to linear mixed models with random intercepts adjusting for age, sex, and BMI. * *p* < 0.05, ** *p* < 0.01. *n* = number of subjects, k = total number of observations. TG, triglycerides; SBP, systolic blood pressure; DBP, diastolic blood pressure; WC, waist circumference; HbA1c, hemoglobin A1c; hsCRP, high-sensitivity C-reactive protein; VAT, visceral adipose tissue; TC, total cholesterol; HDL, high-density lipoproteins; TC, total cholesterol; LDL, low-density lipoproteins.

Several inverse and direct associations between self-reported intake of ACN-containing food groups and cardiometabolic risk factors were observed (Figure 1). For example, intake of berries, dairy products with berries, and ACN-containing vegetables had inverse associations with visceral adipose tissue volume, while wine had direct associations with total cholesterol, HDL-C, and systolic blood pressure. Other direct associations were found between the intake of berries and hemoglobin A1c, and between ACN-containing drinks with hsCRP.

### 3.3. Metabolome Biomarkers Associated with Total ACN Intake

**Figure 2:**
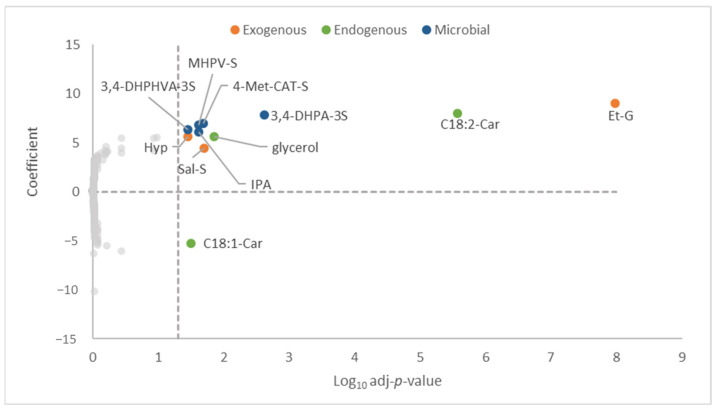
Association between metabolome biomarkers and total intake of ACNs. Censored regression for panel data adjusting for age, sex, and BMI (*n* = 624, k = 1351). *p*-values were calculated and adjusted by Benjamini–Hochberg procedure. Adjusted *p*-values < 0.05 were considered statistically significant. Hyp, hypaphorine; Sal-S, salsolinol sulfate; Et-G, ethyl glucuronide; 4-Met-Cat-S, 4-methylcatechol sulfate; MHPV-S, 4′-hydroxy-3′-methoxyphenyl-γ-valerolactone sulfate; 3,4-DHPHVA-3S, 5-(4-hydroxy(3,4-dihydroxyphenyl)-valeric acid sulfate; 3,4-DHPA-3S, 3,4-dihydroxyphenylacetic acid sulfate; IPA, indolepropionic acid; C18,2-Car, linoleoyl carnitine; C18,1-Car, oleoyl carnitine.

In censored regression analysis, 10 metabolites were positively associated with total ACN intake (Figure 2). Among them, three were exogenous metabolites, hypaphorine, salsolinol sulfate, and ethyl glucuronide, two were endogenous metabolites, including linoleoyl carnitine and glycerol, and five were gut microbial metabolites, 4-methylcatechol sulfate, 4′-hydroxy-3′-methoxyphenyl-γ-valerolactone-sulfate (MHPV-S), 5-(4-hydroxy(3,4-dihydroxyphenyl)-valeric acid sulfate (3,4-DHPHVA-S), 3,4-dihydroxyphenylacetic acid sulfate (3,4-DHPA-3S) and indolepropionic acid. On the other hand, only oleoyl carnitine, another endogenous metabolite, was inversely associated with total ACN dietary intake.

### 3.4. Metabolome Biomarkers Associated with Intake of ACNs Related to Different ACN Dietary Sources

**Figure 3:**
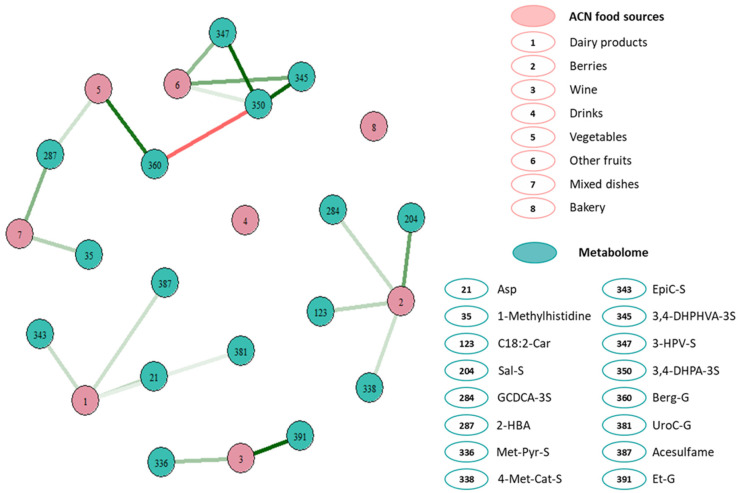
First-order neighborhood of ACNs intake related to different self-reported food groups with plasma metabolome biomarkers according to Mixed Graphical Models in the DCH-NG MAX study (*n* = 624, k = 1351). Edge intensity reflects the strength of the association from strong direct (dark green) to strong inverse association (dark red). Variables included in the mixed graphical model were ACN intake related to self-reported intake of dairy, berries, wines, non-alcoholic drinks (smoothies and fruit juices), vegetables, other fruits, mixed dishes, and bakery, and all the 408 plasma metabolites quantified with our targeted metabolomics method. *n* = number of subjects, k = total number of observations. For a detailed list of foods within each category go to Supplementary Table S1. Asp, asparagine; EpiC-S, epicatechin sulfate; UroC-G, urolithin C-glucuronide; GCDCA-3S, glycochenodeoxycholic 3-sulfate; Sal-S, salsolinol sulfate; 4-Met-Cat-S, 4-methylcatechol sulfate; C18:2-Car, linoleoyl carnitine; 2-HBA, 2-hydroxybenzoic acid; Berg-G, bergaptol glucuronide; Met-Pyr-S, methylpyrogallol sulfate; 3-HPV-S, 5-(3′-hydroxyphenyl)-γ-valerolactone 3’-sulfate; 3,4-DHPHVA-S, 5-(4-hydroxy(3,4-dihydroxyphenyl)-valeric acid sulfate; 3,4-DHPA-3S, 3,4-dihydroxyphenylacetic acid sulfate; Et-G, ethyl glucuronide.

MGM analysis showed associations between self-reported ACN intake from different dietary sources and 16 metabolites (Figure 3). ACNs derived from dairy products were associated with plasma asparagine, epicatechin sulfate, urolithin C-glucuronide, and acesulfame K. ACNs from the intake of berries were associated with linoleoyl carnitine, salsolinol sulfate, glycochenodeoxycholic-3-sulfate (GCDCA-3S) and 4-methylcatechol sulfate. ACNs from wine consumption were linked with methylpyrogallol sulfate (Met-Pyr-S) and ethyl glucuronide. ACNs from vegetable intake were associated with 2-hydroxybenzoic acid and bergaptol glucuronide. ACNs from other fruits were associated with 3,4-DHPHVA-3S, 5-(3′-hydroxyphenyl)-γ-valerolactone 3’-sulfate (3-HPV-S) and 3,4-dihydroxyphenylacetic acid sulfate (3,4-DHPA-3S). Lastly, the consumption of ACNs from mixed dishes was associated with 1-methylhistidine and 2-hydroxybenzoic acid. Overall, not all the metabolites selected in the MGM analysis were related to ACNs or its microbial metabolites, but to other food components such as acesulfame K, ethyl glucuronide, etc. Therefore, metabolome biomarkers were selected considering both statistical analyses, censored regression, and MGM, to be used for the study of its association with cardiometabolic risk factors.

### 3.5. Associations between Selected ACN-Related Metabolome Biomarkers and Cardiometabolic Risk Factors

**Figure 4:**
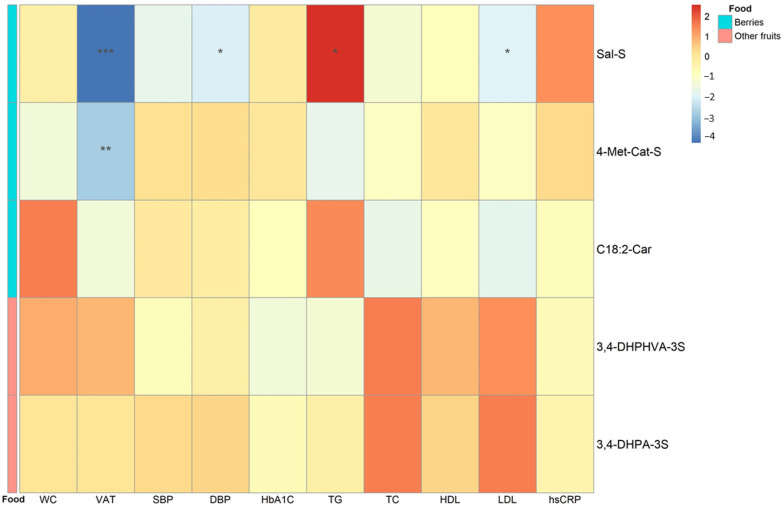
Association between ACN-related selected metabolites and cardiometabolic risk factors in the DCH-NG MAX study (*n* = 624, k = 1351). Standardized coefficients according to linear mixed models with random intercepts adjusting for age, sex, and BMI. Foods associated with the metabolites according to MGM analysis are displayed by colors in the food column. * *p* < 0.05, ** *p* < 0.01, *** *p* < 0.001. *n* = number of subjects, k = total number of observations. Sal-S, salsolinol sulfate; 4-Met-Cat-S, 4-methylcatechol sulfate; C18:2-Car, linoleoyl carnitine; 3,4-DHPHVA-3S, 5-(4-hydroxy(3,4-dihydroxyphenyl)-valeric acid sulfate; 3,4-DHPA-3S, 3,4-dihydroxyphenylacetic acid sulfate; TG, triglycerides; SBP, systolic blood pressure; DBP, diastolic blood pressure; WC, waist circumference; HbA1c, hemoglobin A1c; hsCRP, high-sensitivity C-reactive protein; VAT, visceral adipose tissue; TC, total cholesterol; HDL, high-density lipoproteins; TC, total cholesterol; LDL, low-density lipoproteins.

Metabolites associated with ACN intake in both of the previous analyses were: salsolinol sulfate, 4-methylcatechol sulfate, linoleoyl carnitine, 3,4-DHPHVA-3S, and 3,4-DHPA-S. Figure 4 shows the associations between these metabolites and cardiometabolic risk factors. Out of the metabolites associated with berries’ ACNs, salsolinol sulfate and 4-methylcatechol sulfate were inversely associated with visceral adipose tissue volume. In addition, inverse associations were also found between salsolinol sulfate and LDL-C and diastolic blood pressure. Conversely, there was a direct association between salsolinol sulfate and triglyceride levels (Figure 4). Linoleoyl carnitine, 3,4-DHPHVA-3S, 3,4-DHPA-S did not show any statistically significant association with cardiometabolic risk factors.

## 4. Discussion

The present study shows for the first time the specific associations between ACNs related to different dietary sources, and plasma metabolome biomarkers and their association with cardiometabolic risk factors in a free-living population. These results may take into account not only the quantitative and qualitative heterogeneity of ACNs presence in foods but also the internal dose of specific microbial metabolites generated from ACNs which could have been affected by the food matrix. Indeed, food matrices have been shown to influence the microbial metabolism of (poly)phenols [24]. Ultimately, we observed different associations between ACN-related metabolites and cardiometabolic risk factors in relationship with specific foods suggesting a stronger cardiometabolic benefit associated with the consumption of berries.

Up to 80% of the total intake of dietary ACNs came from the consumption of berries, wines, and non-alcoholic drinks in this observational study. Minor contributors were dairy foods, other fruits, and vegetables. While the MGM analysis revealed different metabolomic fingerprints associated with different dietary sources of ACNs, the resultant metabolites were not specific to ACNs. Therefore, we selected metabolites that were also significantly associated with the censored regression analysis. This was a strict criterion but in the context of such low levels of ACN intake in the overall population (median 1.6 mg/day), it is justified. After applying this selection criterion, only metabolites related to ACNs from berries and other fruits (according to MGM) were tested for their association with cardiometabolic risk factors. Metabolites specifically related to ACNs from other major food sources, such as wines, were excluded. Nonetheless, other studies showed that for example 4-methylcatechol sulfate was increased after a 15-day moderate red wine intervention trial [25]. Therefore, we cannot be fully certain that in our study the same metabolites could be related to other ACN dietary sources. Future randomized controlled trials using single foods are warranted to validate the present results.

Regarding the association between metabolome biomarkers and cardiometabolic risk factors, 4-methylcatechol sulfate showed an inverse association with visceral adipose tissue volume. According to our MGM analysis, 4-methylcatechol sulfate was associated with the intake of ACNs from berries. Similarly, another metabolite associated with ACNs from berries was salsolinol sulfate. Salsolinol sulfate is an alkaloid that has been suggested as a biomarker of banana intake [26]. However, salsolinol can be produced endogenously through dopamine oxidative metabolism [27,28] and may have a role in modulating dopamine neurons activity in the striatum region of the brain [21]. In fact, patients with obesity showed impaired dopamine brain activity, underscoring a potential role for low dopamine activity in obesity (lower reward associated with food intake) [29]. Hence, we speculate that the inverse association between salsolinol sulfate and visceral adipose tissue could be mediated by brain dopamine activity. An animal study showed that a blackberry extract intervention reversed the effects of a high-fat diet increasing dopamine turnover in the brain striatum region [30]. The role of berries on brain dopamine metabolism should be further studied. On the other hand, the other selected metabolites were not associated with cardiometabolic risk factors.

The median value of total ACN intake in the study was 1.6 mg/day, and such intake may not have been high enough to detect metabolome biomarkers found in randomized controlled trials (RCT) with ACN-rich foods [31,32,33]. Many short or long-term RCTs were conducted with capsulated ACNs or berries to discover biomarkers of ACNs intake. In these trials, daily intakes of ACNs typically varied from 100 to 300 mg as single dose intakes [33,34], or between 50–350 mg/day for four weeks [35,36,37]. In general, many parent ACNs and up to 70 phenolic compounds resultant of the gut microbial metabolism of ACNs have been identified [35,36]. Even though the majority of these metabolites were not identified in our study, 4-methylcatechol sulfate, 3,4-DHPHVA-3S, and 3,4-DHPA-3S had been previously associated with ACN intake. Maybe, longer half-lives of these metabolites vs. the others, or the competition of polyphenol substrates for bacteria able to metabolize them limited the production of ACNs metabolites under the low levels of ACN intake (exposure) in the study.

Among the strengths of this study are its observational nature and the fact that dietary data were assessed with 24-HDRs instead of food frequency questionnaires. This last characteristic allowed us to have exact intake data both in terms of amounts and specific food items compared to food frequency questionnaires. However, this also brings the limitation of measurement errors in estimating ACN intake and the short time period surveyed (one 24-HDR at each evaluation time). Another limitation was that the median consumption of dietary ACNs within the population of the DCH-NG MAX study was 1.6 mg/day, which was considerably lower than other studies in which the median intake varied between 9.3 to 52.6 mg/day [38,39,40,41]. This fact could have limited the number of plasma metabolites associated with dietary ACNs. Furthermore, the mean fasting time of the participants at the time the blood samples were drawn was 5 h and the impact of fasting on serum metabolome is uncertain. Nonetheless, this is the first study evaluating the impact of ACNs coming from different dietary sources on plasma metabolome and therefore our results cannot be contrasted with others. While berries contain other polyphenols in addition to ACNs, further research is needed to fully understand the individual and combined effects of different polyphenols of berries on health outcomes. Our approach to isolating the effects of ACNs from berries was conducted from a bioinformatic approach and a more precise study testing the effects of isolated ACNs from berries should corroborate our results. While berries contain other polyphenols in addition to ACNs, further research is needed to fully understand the individual and combined effects of different polyphenols of berries on health outcomes. Our approach to isolating the effects of ACNs from berries was conducted from a bioinformatic approach and a more precise study testing the effects of isolated ACNs from berries should corroborate our results. Last, it is not clear if the microbial metabolites were exclusively related to the ACNs from the dietary source pointed out in the MGM analysis or could have been also produced from ACNs coming from other foods, or even from food components other than ACNs (e.g., other polyphenols apart from ACNs). Although MGM models adjust every association for all the other variables included in the analysis, these sources of confounding cannot be ruled out.

In conclusion, this study shows that the metabolomic fingerprint of ACN consumption depended on its dietary sources. Metabolites associated with the consumption of berries’ ACNs showed inverse associations with visceral adipose tissue. Future RCTs should validate the importance of these foods for cardiometabolic health and their potential mechanisms of action.

## Figures and Tables

**Table 1:** Sociodemographic and clinical characteristics according to tertiles of total ACN intake.

	All *n* = 624 k = 1351	Tertile 1 <0.3 mg ACN/Day k = 453	Tertile 2 0.3–8.9 mg ACN/Day k = 448	Tertile 3 >8.9 mg ACN/Day k = 450
Age (years)	44.7 ± 12.3	43.7 ± 12.6	44.2 ±12.5	46.1 ± 11.8
Gender, female (*n*, %)	745 (55)	236 (52)	250 (55)	256 (57)
BMI (kg/m^2^)	25 ± 4	25 ± 4	24 ± 3	25 ± 4
WC (cm)	87.5 ± 12.1	88.7 ± 12.4	86.0 ± 11.6	87.7 ± 12
VAT (L)	1.3 (0.7–2.5)	1.5 (0.8–2.6)	1.2 (0.6–2.1)	1.4 (0.8–2.5)
Physical activity (*n*, %)				
Not regular	114 (17)	41 (19)	25 (12)	37 (18)
Once/month last 6 months	52 (89	15 (79	22 (11)	12 (6)
Once/month last 12 months	510 (75)	162 (74)	159 (77)	152 (76)
Smoking status (*n*, %)				
Never	353 (52.2)	114 (52.3)	117 (56.89	97 (48.3)
Former	186 (27.5)	53 (24.3)	53 (25.79)	69 (34.39)
Current	137 (20.3)	51 (23.4)	36 (17.59)	35 (17.4)
SBP (mmHg)	117 ± 16	117 ± 15	116 ± 15	116 ± 16
DBP (mmHg)	81 ± 11	80 ± 10	79 ± 10	80 ± 11
HbA1c (mmol/mol)	34.5 ± 6	34.6 ± 7	33.8 ± 5	34.6 ± 6
TG (mmol/L)	1.1 (0.8–1.6)	1.1 (0.8–1.7)	1.0 (0.8–1.4)	1.1 (0.8–1.6)
TC (mmol/L)	4.9 ± 1.0	4.9 ± 0.9	4.9 ± 0.9	5.1 ± 1.0
HDL (mmol/L)	1.6 ± 0.4	1.5 ± 0.4	1.6 ± 0.4	1.6 ± 0.5
LDL (mmol/L)	3.0 ± 0.9	3.0 ± 0.9	2.9 ± 0.9	3.1 ± 0.9
hsCRP (mg/L)	0.7 (0.3–1.6)	0.8 (0.3–1.6)	0.7 (0.3–1.5)	0.7 (0.3–1.6)

BMI, body mass index; WC, waist circumference; VAT, visceral adipose tissue; SBP, systolic blood pressure, DBP, diastolic blood pressure; HbA1c, hemoglobin A1c; TG, triglycerides; TC, total cholesterol; HDL, high-density lipoproteins; LDL, low-density lipoproteins; hsCRP, high-sensitivity C-reactive protein. Variables following a normal distribution are shown as mean ± SD, and those with a skewed distribution are shown as median (p25–p75).

## Data Availability

Data may be available upon request to the Danish Cancer Society (contact: dchdata@cancer.dk).

## Supplementary Materials

The following supporting information can be downloaded at: https://www.mdpi.com/article/10.3390/nu15051208/s1. Table S1. ACN-containing food list in the DCH-NG MAX study, Table S2. Dietary characteristics of the MAX study population according to tertiles of dietary ACN intake.
