# Supplementary material for: Dietary Sources of Anthocyanins and Their Association with Metabolome Biomarkers and Cardiometabolic Risk Factors in an Observational Study
Source: Nutrients. 2023 Feb 28;15(5):1208. doi: 10.3390/nu15051208 (PMC10005166; doi:10.3390/nu15051208)
Supplement: Supplementary file 1 [file nutrients-15-01208-s001.zip › Supplementary table 2.pdf]

Supplementary Table S2. Dietary characteristics of the MAX study population according to tertiles of dietary ACN intake.

	All<br>n= 624<br>k=1,353	Tertile 1<br><0.29 mg ACN/day	Tertile 2<br>0.29-8.9 mg ACN/day	Tertile 3<br>>8.9 mg ACN/day
**Dietary characteristics**				
Energy (10^3 kcal/d)***	2.1 ±0.8	1.9 ±0.8	2.1 ±0.8	2.2 ±0.8
SFA (g/d)*	27 ±15	25 ±15	28 ±15	28 ±16
MUFA (g/d)***	29 ±16	27 ±16	31 ±16	30 ±16
PUFA (g/d)**	14 ±7	13 ±8	15 ±7	14 ±7
Total sugars (g/d)***	73 ±50	58 ±42	77 ±46	73 ±50
Sucrose (g/d)*	35 ±32	31 ±31	37 ±34	37 ±35
Protein (g/d)*	88 ±40	83 ±39	88 ±36	91 ±44
Dietary fiber (g/d)**	22 ± 11	20 ± 11	24 ± 11	22 ± 11
Alcohol (g/d)***	0 (0 - 13.2)	0 (0-0)	0 (0-9.3)	9.5 (0-28.5)
Sodium (g/d)	3.04 ± 1.7	2.96 ±1.8	3.09 ±1.6	3.03 ±1.7
**Food intake**				
Cereal whole grain (g/d)	115 (45-200)	105 (35-205)	125 (60-213)	110 (45-190)
Cereal refined (g/d)	0 (0-80)?	0 (0-90)	0 (0-80)	0 (0-61)
Fruits (g/d)***	150 (0-313)	0 (0-120)	150 (0-250)	110 (0-229)
Berries (g/d)***	0 (0-10)	0 (0-0)	0 (0-6.5)	10 (0-35)
Vegetables (g/d)	140 (30-290)	190 (60-360)	210 (60-394)	247 (98-400)
Legumes (g/d)	0 (0-0)	0 (0-0)	0 (0-0)	0 (0-0)
Nuts and seeds (g/d)	0 (0-10)	0 (0-1.5)	0 (0-15)	0 (0-15)

SFA: Saturated fatty acids; MUFA: monounsaturated fatty acids; PUFA: polyunsaturated fatty acids. *****p for trend<0.05, ** p for trend<0.01, *** p for trend<0.001 using age- and sex-adjusted generalized linear models. Variables with skewed distribution were log-transformed before entering the analyses.
